# Estratégias de Proteção Esofágica para Ablação de Fibrilação Atrial: Resultados Comparativos de Avaliações Endoscópicas Consecutivas

**DOI:** 10.36660/abc.20230913

**Published:** 2025-03-18

**Authors:** Alberto Pereira Ferraz, Cristiano Faria Pisani, Esteban Wisnivesky Rocca Rivarola, Tan Chen Wu, Francisco Carlos da Costa Darrieux, Rafael Alvarenga Scanavacca, Muhieddine Omar Chokr, Carina Abigail Hardy, Sissy Lara de Melo, Denise Tessariol Hachul, Beatriz Hachul de Campos, Mauricio Ibrahim Scanavacca

**Affiliations:** 1 Hospital das Clínicas Faculdade de Medicina Universidade de São Paulo São Paulo SP Brasil Instituto do Coração do Hospital das Clínicas da Faculdade de Medicina da Universidade de São Paulo,São Paulo. SP – Brasil; 2 Hospital Israelita Albert Einstein São Paulo SP Brasil Hospital Israelita Albert Einstein, São Paulo. SP – Brasil

**Keywords:** Fístula Esofágica, Fibrilação Atrial, Ablação por Cateter, Endoscopia

## Abstract

**Fundamento:**

A ocorrência de fístula atrioesofágica após o procedimento ode ablação de fibrilação atrial (FA) continua uma preocupação. Não existe uma abordagem padronizada para minimizar seus riscos e mortalidade.

**Objetivos:**

Apresentar a experiência de sete anos de um monitoramento endoscópico sistemático de lesão esofágica após a ablação de FA por cateter.

**Métodos:**

Estudo unicêntrico retrospectivo de avaliação endoscópica sistemática após ablação e FA em procedimentos consecutivos realizados entre 2016 e 2022. Um valor de p < 0.05 foi considerado estatisticamente significativo.

**Resultados:**

Foram analisados 823 procedimentos de ablação de FA com Esofagogastroduodenoscopia (EGD) de controle. A maioria (n=588; 71,4%) dos pacientes submetidos ao procedimento era do sexo masculino, 575 (69,9%) apresentaram FA paroxística. Monitoramento da temperatura esofágica foi realizada usando um sensor único em 310 pacientes (40,3%) e uma sonda multissensor em 306 (39,8%) pacientes. As lesões estavam presentes em 217 EGD (26,5%): hematoma-equimose em 27 (3,3%), eritema em 14 (1,7%), erosão em 78 (9,5%) e úlcera em 67 (8,2%) pacientes. Nenhuma estratégia de proteção do esôfago foi associada à maior ocorrência de úlceras, com exceção do uso de cateter de ponta de 8mm (14,7% de úlceras com cateter de ponta de 8mm vs. 6,7% com outros cateteres, p = 0,001). Lesões térmicas foram detectadas precocemente e tratadas. A maioria das lesões foi considerada curada na endoscopia, mas um paciente que foi submetido ao isolamento da veia pulmonar com um cateter de ponta de 8mm apresentou fístula esofágica, que foi tratada com sucesso com clipe metálico endoscópico e técnica endoloop.

**Conclusão:**

A incidência de lesões esofágicas é alta na EGD de rotina realizada após a ablação de FA, embora, na maioria dos casos, sua cura ocorra espontaneamente. Pacientes que se submeteram à ablação com o cateter de ponta de 8mm apresentaram lesões térmicas mais graves. Endoscopia esofágica precoce pode ajudar no diagnóstico de lesões em fases iniciais e a prevenção de fístulas após a ablação de FA.

## Graphical abstract

**Figura Central:**
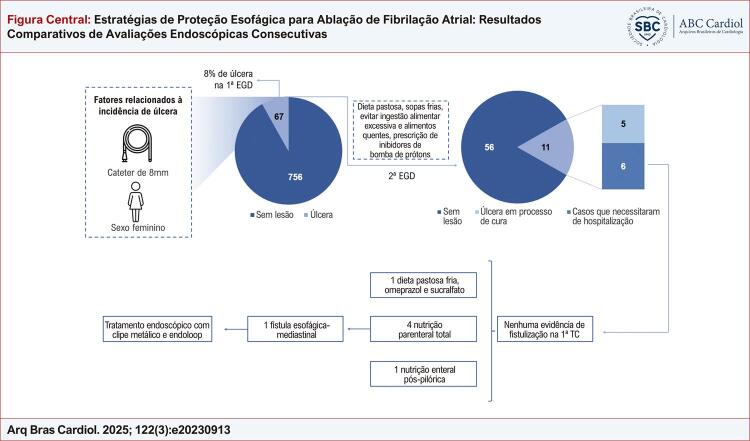
: Estratégias de Proteção Esofágica para Ablação de Fibrilação Atrial: Resultados Comparativos de Avaliações Endoscópicas Consecutivas

Principais resultados de avaliações endoscópicas consecutivas após ablação de fibrilação atrial; a endoscopia esofágica precoce pode ajudar no diagnóstico de lesões esofágicas precoces e prevenir a formação de fístula atrioesofágic.; EGD: Esofagogastroduodenoscopia.

## O que há de novo?

- Diferentes ferramentas e estratégias usadas para ablação da fibrilação atrial apresentam riscos singulares de lesão esofágica;
- A esofagogastroduodenoscopia precoce ajuda no rastreamento de lesão esofágica e permite a identificação da real incidência dessas lesões;
- A detecção precoce de lesões esofágicas graves permite seu tratamento precoce, e o monitoramento de sua evolução pode prevenir a formação de fístula.

## Introdução

A ablação por cateter é um tratamento eficaz e bem estabelecido para restaurar o ritmo sinusal na fibrilação atrial (FA) e se tornou um procedimento amplamente realizado no mundo.^1^ No entanto, a fístula atrioesofágica (FAE), embora rara, continua uma das complicações mais sérias e potencialmente fatais, ocorrendo em até 0,11% dos procedimentos de ablação.^2,3^

Várias estratégias foram implementadas para prevenir lesões esofágicas,^4^ incluindo o uso de sondas esofágicas de sensor único ou multissensores,^5^ estratégias alternativas de fornecimento de energia tais como ablação de alta potência e curta duração,^6^ deslocamento do esôfago utilizando dispositivos especializados,^7^ e monitoramento esofágico de rotina por Esofagogastroduodenoscopia (EGD).^8^ Apesar desses esforços, ainda não existe um consenso sobre a melhor abordagem para detectar lesões esofágicas assintomáticas que poderiam progredir para FAE.^9^

Este estudo tem como objetivo determinar a incidência de lesão esofágica assintomática detectada durante endoscopia pós-ablação e avaliar se estratégias específicas, incluindo o uso de cateteres de 8mm, ou características estão associadas com uma incidência mais baixa dessas lesões (Figura Central).

## Métodos

Este registro compila dados de todos os procedimentos de ablação de FA realizados em um centro de cardiologia terciário entre 2016 e 2022, incluindo pacientes que se submeteram à ablação usando cateteres de ponta de 8 mm. Esse período reflete a introdução da endoscopia pós-procedimento. Características clínicas e ecocardiográficas, estratégias de proteção do esôfago, e achados da EGD foram coletados dos prontuários médicos.

### Estratégia de ablação

Todos os procedimentos foram realizados com o paciente sob anestesia geral. Um ecocardiograma transesofágico (ETE) foi realizado antes da ablação para detecção de trombo no átrio esquerdo (AE). Pacientes do sistema de saúde público, a quem o mapeamento eletroanatômico não estava disponível, foram submetidos somente à fluoroscopia – ou usando-se um cateter de ablação da veia pulmonar (pulmonary vein ablation catheter – PVAC), ou por crioablação, ou por ablação circunferencial (guiada por venograma e cateter e mapeamento circular, e usando um cateter de 8mm). O desfecho do procedimento foi isolamento da veia pulmonar em todos os casos, além de outras lesões como isolamento da parede posterior, istmo mitral e homogeneização da cicatriz, de acordo com a decisão do operador. Todos os procedimentos foram realizados por cinco operados diferentes. Quando se utilizou um cateter de ponta de 8mm, o procedimento de ablação foi realizado sob temperatura (55^o^C) e potência (até 50W na projeção anterior e até 30W na projeção posterior) controladas. A ablação por cateter com ponta irrigada foi realizada sob potência controlada, inicialmente pela técnica de arrasto (*dragging*) com aplicação de 30W na parede anterior e 20W na parede posterior. Após 2018, a tecnologia SURPOINT tornou-se disponível, e lesões de ablação com 40W foram realizadas ponto a ponto visando o índice de 550 na parede anterior e 400 na parede posterior.

Quando ocorria algum aumento na temperatura esofágica, a aplicação de radiofrequência (RF) foi interrompida, a capacidade foi reduzida para 20W ou 15W (de acordo com o caso), e a RF foi retomada somente após normalização da temperatura. Quando lesões SURPOINT ponto a ponto foram usadas, o tempo de aplicação foi diminuído visando um índice de 300. Quando uma sonda linear foi usada, a posição do sensor foi guiada por fluoroscopia para posicionar o sensor mais perto da área de ablação. Foi utilizada uma sonda de ETE para deslocar o esôfago, se sua posição estivesse próxima à veia. Em alguns casos, a temperatura esofágica foi monitorada usando uma estratégia de deslocamento do esôfago. O sistema multieletrodo utilizado foi o Circa, com o alarme definido em duas temperaturas, 37,5ºC (quente) e 38ºC (risco), no início do procedimento. Se ocorresse um aumento repetido da temperatura esofágica, a RF era aplicada com curta duração (5- 10s) até se alcançar o isolamento da veia pulmonar.

### Esofagogastroduodenoscopia

A EGD foi realizada em até sete dias após a ablação, sendo a maioria no dia seguinte ao procedimento de ablação. As lesões observadas na EGD após a ablação foram categorizadas de acordo com o sistema de Classificação Kansas City (KCC),^10^ com descrição adicional de lesões e traumas, e ênfase em lesões do tipo KCC 2b (úlceras), dado seu risco mais alto de progressão à perfuração e fístula. Nos casos de lesão na primeira EGD, o paciente recebia alta e era orientado a manter o uso de Inibidores de Bomba de Prótons (IBP) e sucralfato. Uma segunda EGD foi recomendada após sete dias em pacientes que apresentavam lesões mais graves (KCC 2b). Nossa estratégia de seguimento após ablação e EGD foi publicado previamente.^4^

### Análise estatística

Os dados contínuos foram apresentados em média e desvio padrão ou mediana e intervalo interquartil em caso de distribuição assimétrica. Os dados categóricos foram apresentados como frequência absoluta e porcentagens. A ocorrência de úlceras foi descrita de acordo com cada característica qualitativa, primeiramente em uma análise bivariada para examinar a relação entre duas variáveis. A associação foi verificada por teste do qui-quadrado ou teste de razão de verossimilhança, enquanto as características quantitativas foram descritas de acordo com a ocorrência de úlcera, e comparadas usando o teste t de Student não pareado ou o teste de Mann-Whitney de acordo com a distribuição da probabilidade dos dados. O modelo conjunto (*joint model*) foi testado por regressão logística múltipla com as características que apresentaram um nível descritivo abaixo de 0,20 na análise bivariada (p<0,20). A ocorrência de úlcera foi descrita em cada endoscopia em pacientes que repetiram o exame e verificaram a mudança na ocorrência de úlcera entre os exames pelo teste de McNemar.

O programa IBM-SPSS para Windows, versão 22.0, foi usado para as análises, e o programa Microsoft Excel 2013 foi usado para tabular os dados. Os dados foram testados usando o teste de normalidade de Shapiro-Wilk. Um nível de significância de 5% foi aplicado nos testes.

## Resultados

### Pacientes e características do procedimento

As características dos pacientes estão apresentadas na Tabela 1. Um total de 823 procedimentos de ablação com EGD controlada foram realizados em nossa instituição entre janeiro de 2016 e dezembro de 2022. A maioria dos pacientes era do sexo masculino, com idade mediana de 60 anos, e a maioria apresentou FA paroxística. Os valores médios do diâmetro do AE e da fração de ejeção ventricular esquerda foram 42,3±6,3mm e 61±8%, respectivamente.

**Tabela 1:** – Características dos pacientes e do procedimento de ablação

	Total
**Pacientes**	823
**Sexo masculino**	588 (71,4)
**Idade (anos)**	60 (52, 68)
**Classificação da FA**	
Paroxística	575 (69,9)
Persistente	248 (30,1)
**Escore CHA2DS2-VASc**	1,66±1,39
**Diâmetro AE (mm)**	42,3 ± 6,3
**Volume AE (mL/m^2^)**	39,5 ± 12,8
**FEVE, %**	61,3 ± 8,5
**Cardioversão elétrica prévia**	189 (22,9)
**Ablação de FA prévia**	147 (17,8)
**Mapeamento eletroanatômico**	655 (79,6)
**Abordagem de ablação**	
Fluoroscopia	168 (20,4)
Carto 3	601 (73)
Ensite	53 (6,4)
Microport	1 (0,1)
**Estratégia de ablação**	
50/30W, 55T	150 (18,2)
Dragging 30W	332 (40,3)
HPSD	40 (4,9)
SurPoint	261 (31,7)
Crioablação	6 (0,7)
PVAC	34 (4,1)
**Cateter**	
8mm	149 (18,1)
ThermocoolST	135 (16,4)
STSF	469 (57)
Tacticath	27 (3,3)
Flexability	2 (0,2)
ArcticFront	6 (0,7)
PVAC Gold	34 (4,1)
3DFireMagic	1 (0,1)
**Proteção esofágica**	
Nenhum dispositivo	24 (3,1)
Sonda linear	310 (40,3)
Sonda multieletrodo	306 (39,8)
Sonda de ETE	122 (15,9)
Deslocamento do dispositivo	7 (0,9)
**Duração do procedimento (minutes)**	210 (180; 260)

Valores em número (%), média ± desvio padrão ou mediana (p25–p75); AE: atrial esquerdo; FA: fibrilação atrial; FEVE: fração de ejeção do ventrículo esquerdo; ETE: ecocardiograma transesofágico; PVAC: pulmonary vein ablation catheter; STSF: Thermocool Smarttouch Surroundflow.

**Figura 1:**
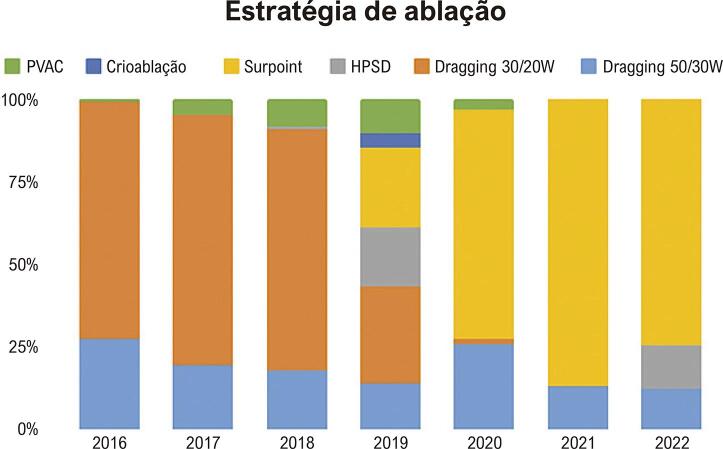
– Estratégia para ablação de fibrilação atrial adotada no InCor ano a ano; houve uma mudança na estratégia utilizada conforme novas tecnologias se tornaram disponíveis (p<0,001). PVAC: pulmonary vein ablation catheter.

O isolamento circunferencial da veia pulmonar foi a abordagem padrão ou com mapeamento eletroanatômico ou guiado por fluoroscopia nos pacientes do sistema público. A maioria (91,8%) dos casos de mapeamento eletroanatômico foi realizado com o sistema Carto e cateteres com sensor de contato, principalmente com a tecnologia Smartouch Surround Flow (STSF). Houve uma mudança na principal estratégia usada para ablação ao longo dos anos, conforme descrito na Figura 1.

**Figura 2:**
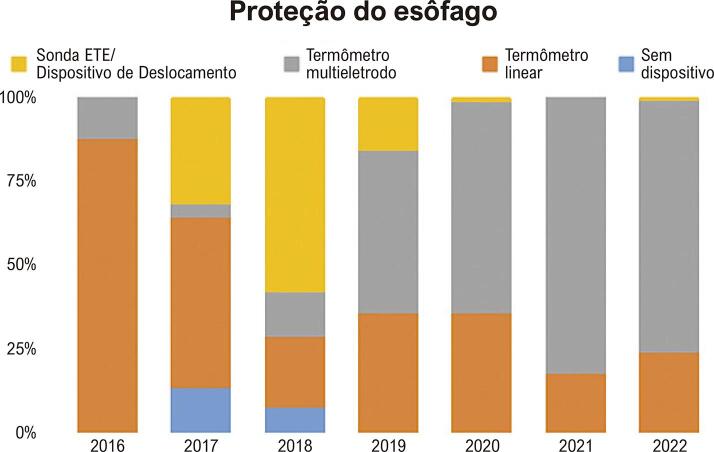
– Distribuição anual da estratégia usada para proteção esofágica na ablação de fibrilação atrial adotada no InCor entre 2016 e 2022 (p<0,001). Nos anos de 2016 e 2017, a estratégia mais usada foi a sonda linear. De 2017 a 2018, iniciamos o deslocamento do esôfago usando a sonda de ecocardiograma que foi abandonada em 2019; subsequentemente, a sonda multieletrodo em forma de S foi a estratégia mais comum.

A estratégia de proteção do esôfago pôde ser aplicada em 769 casos. Uma sonda de sensor único foi a estratégia usada em 40,3% dos casos e a de múltiplos sensores usada em 39,8%. Desvio da sonda transesofágica foi usado em 15,9% dos casos, e deslocamento do dispositivo em 0,9%. Nenhum dispositivo foi usado em somente 3,1% dos casos, em que a potência da RF na parede posterior foi empiricamente diminuída. Houve uma mudança na escolha da estratégia de proteção do esôfago em cada ano, o que está descrito na Figura 2.

### Incidência de lesões esofágicas

Anormalidades agudas foram detectadas em 26,2% dos 823 EGDs. Hematoma e equimose foram encontrados em 3,3% dos pacientes, eritema (KCC1) em 1,7% e erosão (KCC2A) em 9.5%. Lesões esofágicas traumáticas foram encontradas em 3,8% dos pacientes em que a sonda de ETE foi usada para deslocar o esôfago. A incidência de lesões esofágicas após a EGD está descrita na Tabela 2. Em 227 (30,6%) das endoscopias realizadas após a ablação, havia uma quantidade significativas de restos alimentícios, apesar de um período de jejum de oito horas. Tal fato foi atribuído à redução do esvaziamento gástrico secundária a aplicações de RF próximo ao esôfago e ao nervo vago, levando à gastroparesia.

**Tabela 2:** – Lesões endoscópicas detectadas nos pacientes submetidos à esofagogastroduodenoscopia

Sem lesões	606 (73,5)
**Hematoma-Equimose**	27 (3,3)
**Eritema (KCC1)**	14 (1,7)
**Erosão (KCC2A)**	78 (9,5)
**Úlcera (KCC2B)**	67 (8,2)
**Lesão traumática**	31 (3,8)

KCC: Kansas City Classification.

### Preditores de lesões esofágicas

Em uma análise bivariada, não houve diferença de idade, tamanho do AE ou fração de ejeção entre paciente com e sem úlcera. Estratégias protetoras não esofágicas foram correlacionadas com menos lesões esofágicas KCC2B. A mediana da temperatura esofágica mais alta foi similar em ambos os grupos: 38,6 (38; 39,5)ºC nos pacientes sem úlcera e 38,7 (37,8; 40)ºC nos pacientes com úlcera. Entre os vários parâmetros da análise bivariada especificados na Tabela 3, sexo, tamanho do AE, tipo de anticoagulante oral, tipo de cateter, mapeamento eletroanatômico, estratégia de ablação, e proteção do esôfago mostraram um nível descritivo abaixo de 0,20 (p<0,20) e foram subsequentemente incluídos em uma análise de regressão logística múltipla. No modelo de regressão logística, o sexo feminino correlacionou-se com um risco maior de úlcera. O uso de um cateter de ponta de 8mm também aumento o risco de úlcera (Tabela 4).

**Tabela 3:** – Ocorrência de úlcera de acordo com a população estudada e resultados das análises bivariadas

Variável	Sem úlcera	Com úlcera	Valor p
**Idade (anos)**	60 (52; 68)	63 (50; 71)	0,578
**Sexo**			
Masculino	546 (93,5)	38 (6,5)	0,006
Feminino	206 (87,7)	29 (12,3)	
**Escore CHA2DS2-VASc**			0,338
**Classificação da FA**			0,643
Paroxística	526 (92)	46 (8)	0,825
Persistente	226 (91,5)	21 (8,5)	
**Átrio esquerdo (mm)**	42,5 ± 6,3	40,9 ± 5,5	0,065
**Átrio esquerdo (mL/m^2^)**	39,8 ± 12,9	37,1 ± 11,3	0,254
**FEVE (%)**	63 (60; 66)	65 (60; 68)	0,360
**ACO prévio, n (%)**			0,084
**Cardioversão elétrica prévia**			0,504
**Ablação de FA prévia**			0,786
**Temperatura máxima (ºC)**	38,6 (38; 39,5)	38,7 (37,8; 40)	0,705
**Duração do procedimento (minutos)**	210 (180; 260)	210 (180; 270)	0,766
**Cateter, n (%)**			0,037
8mm	127 (85,2)	22 (14,7)	
ThermocoolST	124 (91,9)	11 (8,1)	
STSF	438 (93,3)	31 (6,7)	
Tacticath	27 (100)	0 (0)	
Flexability	2 (100)	0 (0)	
ArcticFront	6 (100)	0 (0)	
PVAC Gold	31 (91,2)	3 (8,8)	
3DFireMagic	1 (100)	0 (0)	
**Mapeamento eletroanatômico, n (%)**	604 (92,8)	47 (7,2)	0,048
**Abordagem de ablação, n (%)**			0,243
Fluoroscopia	148 (88,1)	20 (11,9)	
Carto 3	556 (93)	42 (7)	
Ensite	47 (90,4)	5 (9,6)	
Microport	1 (100)	0 (0)	
**Estratégia de ablação, n (%)**			0,038
50/30W, 55T	127 (85,2)	22 (14,8)	
Dragging 30W	305 (92,1)	26 (7,9)	
HPSD	38 (95)	2 (5)	
SurPoint	245 (94,6)	14 (5,4)	
Crioablação	6 (100)	0 (0)	
PVAC	31 (91,2)	3 (8,8)	
**Proteção esofágica, n (%)**			0,122
Sem dispositivo	21 (87,5)	3 (12,5)	
Sonda linear	279 (90,3)	30 (9,7)	
Sonda multieletrodo	287 (94,4)	17 (5,6)	
Sonda ETE	107 (88,4)	14 (11,6)	
Deslocamento do dispositivo	7 (100)	0 (0)	

Valores em número (%), média ± desvio padrão ou mediana (p25–p75); FA: fibrilação atrial; FEVE: fração de ejeção do ventrículo esquerdo; ETE: ecocardiograma transesofágico; PVAC: pulmonary vein ablation catheter; STSF: Thermocool Smarttouch Surroundflow; ACO: anticoagulante oral.

**Tabela 4:** – Análise multivariada dos principais fatores relacionados à incidência de úlcera esofágica

Variável	OR	IC95%		p
		Inferior	Superior	
**Sexo feminino**	1,82	1,02	3,32	0,041
**Átrio esquerdo (mm)**	0,97	0,92	1,01	0,129
**FEVE (%)**	1,02	0,98	1,06	0,318
**Cateter de 8mm**	3,14	1,21	8,18	0,019

Regressão logística múltipla; FEVE: fração de ejeção do ventrículo esquerdo.

### Seguimento das lesões esofágicas

**Figura 3:**
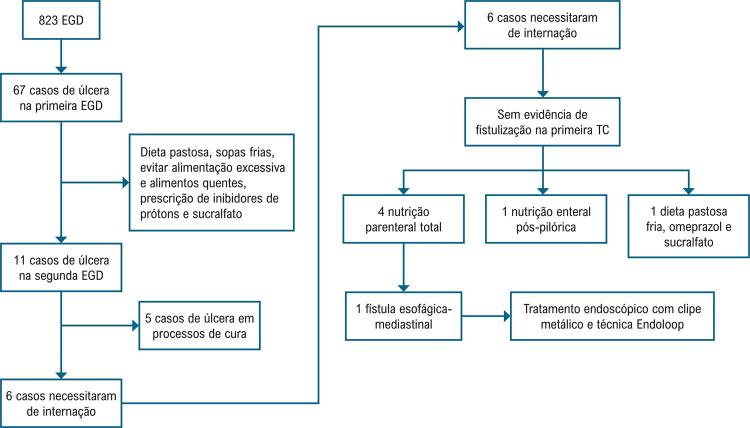
– Evolução dos casos de úlcera. EGD: esofagogastroduodenoscopia; TC: tomografia computadorizada.

Na maioria dos pacientes com lesões traumáticas menores, a EGD foi repetida durante seguimento ambulatorial, e o resultado avaliado pelo médico solicitante. Nos 67 pacientes com úlcera, o tamanho médio da lesão foi 9,6±3,8mm. A lesão foi curada em 57 (83,8%) pacientes, mas 11 (16,2%) pacientes permaneceram com lesão KCC2B. Seis deles (9% dos casos com úlcera) foram readmitidos no hospital, e a primeira Tomografia Computadorizada (TC) não mostrou evidência de fístula nesses pacientes. Quatro dos pacientes foram mantidos em nutrição parenteral total, e um em nutrição enteral por meio de uma sonda esofágica posicionada por endoscopia, e um paciente com dieta pastosa fria, omeprazol e sucralfato. A EGD foi repetida nos pacientes que não apresentaram sinais de fistulização em uma nova TC. Um paciente evoluiu com fístula esofágica-mediastinal 14 dias após a ablação. O procedimento foi conduzido com um cateter de ponta 8mm, guiado por fluoroscopia. O paciente foi tratado endoscopicamente com sucesso, utilizando-se um clipe metálico e técnica endoloop (Figura 3).

## Discussão

As lesões esofágicas ainda constituem um problema durante procedimentos de ablação da FA. Embora raras, com uma incidência inferior a 0,02%, essas lesões requerem medidas de proteção. Nos últimos anos, tecnologias de proteção do esôfago e novas técnicas de ablação têm levado a diferentes riscos do procedimento.^11,12^

Um estudo nacional brasileiro incluiu 10 casos de FAE de oito centros em que se realizaram 8603 procedimentos de ablação entre 2003 e 2015, resultando em uma incidência de 0,113%.^3^ Nessa série, metade dos pacientes apresentaram manifestações clínicas de FAE que foram detectadas por seus médicos. Além disso, sete dos 10 pacientes foram a óbito e somente um paciente teve recuperação completa. Diante desses achados, decidimos que todos os pacientes em nosso grupo que se submeteram à ablação da FA deveriam se submeter ao monitoramento endoscópico a fim de se detectar, precocemente, pacientes em risco e iniciar tratamento precoce quando apropriado.^4^

**Figura 4:**
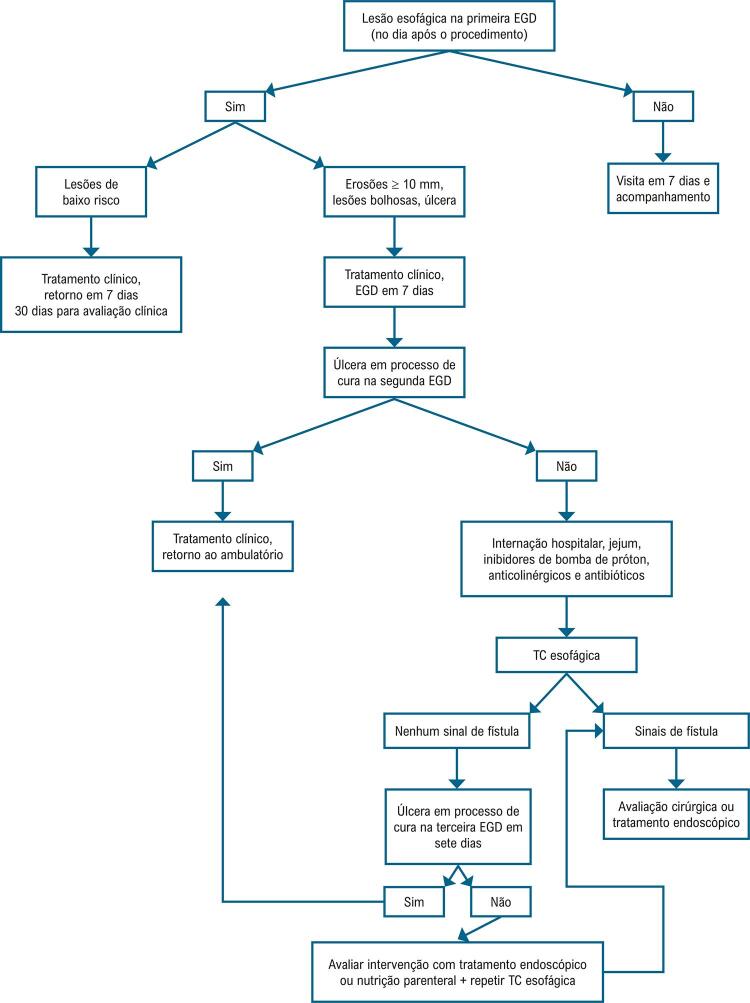
– Protocolo de avaliação e tratamento da lesão esofágica; TC: tomografia computadorizada; EGD: esofagogastroduodenoscopia.

Nós realizamos rotineiramente endoscopia no dia seguinte ao procedimento de ablação de FA. Todos os pacientes, independentemente dos achados endoscópicos, são mantidos em IBPs por 30 dias. Para os pacientes com baixo risco de lesões (eritema e pequenas erosões superficiais), mantemos somente o tratamento com IBP sem uma endoscopia de controle. Em pacientes com erosões maiores que 10 mm, úlceras ou lesões bolhosas, a endoscopia é repetida sedias dias depois. Ainda, adicionamos sucralfato para aumentar a acidez gástrica e esofágica. Quando as úlceras persistem por mais sete dias, os pacientes são internados, mantidos em jejum e IBP endovenoso, e são iniciados antibióticos e drogas anticolinérgicas. É realizada uma TC do esôfago com contraste oral para investigar perfuração do esôfago, e o exame é repetido alguns dias depois, antes de se repetir uma endoscopia com insuflação de CO_2_. Nos pacientes em que a úlcera continuou crescendo e aqueles com sinais e sintomas de perfuração esofágica, é realizada endoscopia terapêutica (Figura 4).^13,14^

Recentemente, nós publicamos nossa série e 677 pacientes com FA que se submeteram à ablação por RF guiada por mapeamento eletroanatômico, PVAC ou crioablação.^9^ Na presente série, nós incluímos esses pacientes, além de 149 pacientes que se submeteram à ablação por RF usando cateteres de ponta de 8mm. Na série anterior, lesões graves (KCC2B) foram encontradas em 6,8% dos pacientes.

Na presente série, úlcera esofágica foi detectada em 67 (8,2%) dos 823 casos. Lesões esofágicas foram curadas na maioria dos casos após uma semana. Seis pacientes foram internados novamente e mantidos em nutrição parenteral, nutrição enteral pós-pilórica ou dieta pastosa segundo protocolo. Uma TC do tórax e uma nova EGD foram realizadas e um paciente evoluiu com fístula esofágica (Figura Central). Esse paciente foi submetido a isolamento da veia pulmonar usando um cateter de 8mm. Após admissão hospitalar, uma TC realizada sete dias após a ablação revelou espessamento parietal e leve densificação de tecido adiposo do mediastino, adjacente ao terço médio do esôfago torácico, sem sinais de pneumomediastino ou fístula. O paciente foi mantido em jejum e com nutrição parenteral. Uma nova TC 14 dias após a ablação revelou uma pequena lesão na parede anterior esquerda do terço médio do esôfago torácico, associada com uma pequena bolha de ar de pneumomediastino próximo à parede posterior do AE. Decidiu-se por realizar tratamento endoscópico com clipes e técnica endoloop.^14^ Essa técnica é indicada para homeostasia em sangramento gastrointestinal e tumores esofágicos; seu uso em caso de fístula após a ablação de FA não está bem documentado na literatura. No entanto, como pudemos observar os primeiros sinais de formação de fístula, e uma vez que o paciente não apresentou febre ou qualquer evidência de infecção sistêmica nos exames de sangue, decidimos por realizar tratamento endoscópico disponível e antibioticoterapia profilática por 14 dias.

Nova TC de controle foi realizada no quarto dia após endoscopia terapêutica. Nutrição enteral foi iniciada no sexto dia. Uma nova TC foi realizada três semanas após a endoscopia sem sinais de fístula. Após se descartar fístula remanescente, a EGD revelou uma retração da cicatriz no esôfago e fechamento da fístula. Em seguida, o paciente iniciou dieta oral e recebeu alta. Não se observaram complicações em um ano de seguimento. Os outros cinco pacientes que estiveram internados tiveram a úlcera curada em EGD sequencial e provavelmente teriam apresentado um pior desfecho se o risco de perfuração não tivesse sido identificado e o tratamento clínico prescrito.

Estudos prévios relataram uma incidência de lesões esofágicas em endoscopia de controle variando entre 1,6%^15^ e 35,5%.^8^ Há várias diferenças entre esses estudos, quanto às estratégias de monitoramento da temperatura esofágica, uso de dispositivos de proteção e a biofísica das aplicações de RF. Yarlagadda et al.^10^ descreveram uma incidência de 570 lesões (15%), 206 (36%) lesões tipo 1 (KCC 1), 222 (39%) lesões tipo 2a (KCC 2A) e 142 (25%) tipo 2b (KCC 2B). Seis dos 142 pacientes com KCC2B (4,2%) evoluíram para tipo 3: cinco era tipo 3a e um era tipo 3b. Halbfass et al.^8^ identificaram uma prevalência de 18% de qualquer lesão e de 6% de lesões ulceradas em pacientes assintomáticos. Diferentemente de Halbfass et al.,^8^ em nossa série, EGD foi sistematicamente realizada, possivelmente melhorando a precisão da detecção de lesões subclínicas, resultando em taxas mais altas de incidência.

Um estudo multicêntrico recente, o estudo POTTER-AF, relatou uma incidência total de fístula de 0,025%.^16^ Contudo, a incidência real deve ser mais alta, uma vez que a maioria dos centros não implementou protocolos de detecção de rotina pós-procedimento, levando a um potencial subdiagnóstico e subrelato.^17,18^ A ausência de casos de FAE detectados nos pacientes entre 1996 e 2003 pode ser atribuída a diferenças nas técnicas de ablação, baixa detecção e insuficiente rastreamento ativo. Em nossa instituição, implementamos medidas direcionadas à identificação de pacientes em risco. No estudo, o número mais alto de casos de FAE relatado por um único centro foi cinco, com uma incidência mínima de 0,4% em um período de tempo específico. Isso destaca a variabilidade na acurácia diagnóstica nos centros participantes. Consistente com os achados de um estudo brasileiro prévio, o estudo POTTER-AF mostrou que somente pacientes com diagnóstico precoce e encaminhamento em tempo para cirurgia sobreviveram.

Esvaziamento gástrico reduzido, evidenciado pela presença de restos alimentares apesar do jejum foi observado em 32% dos casos. Na literatura, alterações na motilidade gástrica sintomáticas, causadas por lesão térmica ao plexo neural periesofágico, foram relatadas em 5% a 74% dos procedimentos. Em alguns casos, uma paralisia transitória, porém grave, pode ocorrer, necessitando de jejum prolongado.^19^

Um dado interessante em nossa série foi o fato de que o sexo feminino se relacionou independentemente com um risco mais alto de lesões esofágicas. Tal fato pode estar relacionado ao achado de que as mulheres geralmente apresentam uma parede atrial esquerda mais fina protegendo o esôfago.

Um fator independente associado com uma incidência mais alta de lesões esofágicas nesta série foi o uso de cateteres de ponta de 8mm (OR 3,14, CI 1,21-8,18; P=0,019). Tal fato provavelmente contribuiu para uma incidência mais alta de lesões esofágicas em comparação à nossa publicação prévia.^9^ Apesar de cateteres de ponta de 8mm tenha sido amplamente substituído por cateteres de ponta irrigada na maioria dos centros, nós continuamos a usá-los uma vez que eles podem ser reprocessados, permitindo a realização de ablação de FA em pacientes no sistema púbico de saúde. Assim, quando esses cateteres são utilizados, é necessária vigilância adicional.

Em nossa série, a abordagem à proteção do esôfago durante a ablação mudou ao longo dos anos. Sondas de sensor único e sondas multissensores de monitoramento foram usadas na maioria dos casos. Até 2018, o desvio do esôfago com uma sonda de ETE foi empregada em mais da metade dos procedimentos; contudo, tal estratégia foi interrompida graças a distorções importantes no mapeamento e o risco de lesões traumáticas.^20^ Atualmente, a sonda multissensor em “S” é nossa principal estratégia de proteção. Estudos anteriores sobre resfriamento esofágico com uma medida de proteção contra a formação de fístula levaram a resultados variados.^21^ Mais recentemente, o estudo IMPACT^22^ demonstrou que estratégias termais de proteção visando reduzir a temperatura esofágica são seguras e podem diminuir significativamente lesões térmicas relacionadas à ablação.

Diferentemente de estudos anteriores,^23^ o uso de pulsos de alta potência e curta duração não se correlacionou com menos lesões esofágicas. Ainda, não se comprovou que o uso de força de contato e tecnologia SurPoint é mais seguro que outras abordagens. Embora novas tecnologias, tais como ablação de campo pulsado sejam promissoras, elas ainda não são disponíveis universalmente. Assim, a preocupação com lesão esofágica continua uma realidade.^24-27^

A principal limitação deste estudo é o fato de se tratar de um registro retrospectivo de dados de uma estratégia implementada em 2016 em nossos pacientes que foram submetidos a uma EGD controle após a ablação de FA por cateter, apesar de se ter observado um aumento da temperatura esofágica. A maioria das primeiras EGDs pôde ser registrada, por haverem sido realizadas no dia seguinte à ablação. Somente pacientes com lesões mais graves foram submetidos à EGD uma semana depois. Observações recentes sugeriram que a endoscopia precoce pode falhar em detectar lesões graves que apareçam nas semanas seguintes. Porém, nenhum dos nossos pacientes retornou ao hospital para tratamento de lesão esofágica, nem apresentou qualquer complicação no seguimento.

Embora o uso de cateter de ponta de 8mm seja considerado uma estratégia “antiga”, ela ainda é empregada em alguns centros. Em nossas séries, seu uso foi associado com um risco mais alto de lesões esofágicas. Opinamos que tal fato não seja uma limitação de nosso estudo, e sim um achado importante que contribui para o conhecimento por parte dos médicos sobre o risco aumentado associado ao uso desses cateteres.

Nossos resultados devem ser interpretados como geradores de hipótese, e requerem maior investigação por meio de estudos com delineamento prospectivo.

## Conclusões

A incidência de lesões esofágicas após a ablação por cateter é alta quando endoscopia de rotina é realizada, porém, na maioria dos casos, a lesão se cura espontaneamente. No entanto, a ablação com 50/30W usando um cateter de ponta de 8mm está relacionada a uma maior ocorrência de úlceras, e é necessário se definir uma melhor estratégia de proteção do esôfago. A endoscopia esofágica precoce pode identificar pacientes em risco de desenvolverem FAE após ablação da FA, e o tratamento clínico precoce parece ser eficaz para prevenir a progressão de desfechos clínicos mais graves.
