# Supplementary material for: Estratégias de Proteção Esofágica para Ablação de Fibrilação Atrial: Resultados Comparativos de Avaliações Endoscópicas Consecutivas
Source: Arq Bras Cardiol. 2025 Mar 18;122(3):e20230913. [Article in Portuguese] doi: 10.36660/abc.20230913 (PMC12013736; doi:10.36660/abc.20230913)
Supplement: Translation

# Translation: Esophageal Protection Strategies for Ablation of Atrial Fibrillation: Comparative Results of Consecutive Endoscopic Evaluation

**DOI:** 10.36660/abc.20230913i

**Authors:** Alberto Pereira Ferraz, Cristiano Faria Pisani, Esteban Wisnivesky Rocca Rivarola, Tan Chen Wu, Francisco Carlos da Costa Darrieux, Rafael Alvarenga Scanavacca, Muhieddine Omar Chokr, Carina Abigail Hardy, Sissy Lara de Melo, Denise Tessariol Hachul, Beatriz Hachul de Campos, Mauricio Ibrahim Scanavacca

**Affiliations:** 1 Brazil Instituto do Coração do Hospital das Clínicas da Faculdade de Medicina da Universidade de São Paulo, São Paulo. SP – Brazil; 2 Brazil Hospital Israelita Albert Einstein, São Paulo, SP – Brazil

**Keywords:** Esophageal Fistula, Atrial Fibrillation, Catheter Ablation, Endoscopy

## Abstract

**Background:**

Atrial-esophageal fistula following AF ablation remains a concern. There is no standardized approach to minimize its risk and morbidity.

**Objective:**

To present the seven-year experience of a systematic endoscopic surveillance of esophageal injury after AF catheter ablation.

**Methods:**

This is a retrospective single center registry of systematic endoscopic evaluation after AF ablation in consecutive procedures performed from 2016 to 2022. A p-value of <0.05 was considered statistically significant.

**Results:**

823 AF ablation with controlled esophagogastroduodenoscopy (EGD) were analyzed. Most patients (n=588, 71.4%) were male, 575 (69.9%) had paroxysmal AF. Esophageal temperature monitoring was performed using a single sensor in 310 patients (40.3%) and a multi–sensor probe in 306 (39.8%). Lesions were presented in 217 EGD (26.5%): hematoma-ecchymosis in 27 (3.3%), erythema in 14 (1.7%), erosion in 78 (9.5%) and ulcer in 67 (8.2%) patients. No esophageal protective strategy was associated with more ulcers, except the use of 8mm tip-catheter (14.7% of ulcers with 8mm tip catheter vs 6.7% with other catheters, p = 0.001). Thermal lesions were early detected and treated. Most lesions were considered healed at endoscopy, but one patient who underwent pulmonary vein isolation with an 8m tip catheter had esophageal fistula, treated successfully with endoscopic metal clip and endoloop technique.

**Conclusion:**

The incidence of esophageal lesions at routine EGD following AF ablation is high, although in most of the cases they heal spontaneously. Patients who underwent ablation with the 8mm tip catheter had more severe thermal lesions. Early esophagus endoscopy may help the diagnosis of early-stage lesions and the prevention of fistula formation after AF ablation.

## What’s New?

- Different tools and strategies used for atrial fibrillation ablation present singular risks of esophageal damage.
- Early esophagogastroduodenoscopy aids in the screening of esophageal injury and allows for the identification of the true incidence of these lesions.
- Early detection of severe esophageal lesions permits their early treatment and monitoring their evolution may avoid esophageal fistula formation.

## Introduction

Catheter ablation is an established and effective treatment for restoring sinus rhythm in atrial fibrillation (AF) and has become a widely performed procedure worldwide.^1^ However, atrioesophageal fistula (AEF), though rare, remains one of the most serious and potentially fatal complications, occurring in up to 0.11% of ablation procedures.^2,3^

Various strategies have been implemented to prevent esophageal lesions,^4^ including the use of single- and multi-sensor esophageal probes,^5^ alternative energy delivery strategies such as high-power/short-duration ablation,^6^ esophageal displacement using specialized devices,^7^ and routine esophageal monitoring with esophagogastroduodenoscopy (EGD).^8^ Despite these efforts, there is still no consensus on the optimal approach to detect asymptomatic esophageal lesions that could progress to AEF.^9^

This study aims to determine the incidence of asymptomatic esophageal injury detected during post-ablation endoscopy and to evaluate whether specific strategies, including the use of 8mm tip catheters or features are associated with a reduced incidence of these lesions (Central Illustration).

## Methods

This registry holds data from all AF ablations performed at a tertiary cardiology center between 2016 and 2022, including patients that underwent ablation using 8mm tip catheters. This period reflects the introduction of post-procedure endoscopy. Clinical and echocardiographic characteristics, esophageal protection strategies, and EGD findings were extracted from medical records.

### Ablation strategy

All procedures were performed with the patient under general anesthesia. A transesophageal echocardiogram (TEE) was performed before the ablation for left atrial (LA) thrombus detection. Patients from the public health system, in which electroanatomical mapping (EAM) was not available, underwent fluoroscopic procedure only – with a pulmonary vein ablation catheter (PVAC), by cryoablation or by circumferential ablation (guided by venogram and circular mapping catheter, and using an 8mm tip catheter). The endpoint of the procedure was pulmonary vein isolation in all cases, in addition to other lesions such as posterior wall isolation, mitral isthmus, and scar homogenization according to operator decision. All the procedures were performed by five different operators. When using an 8mm catheter, ablation was temperature (55ºC) and power (up to 50W on the anterior and 30W on the posterior projections) controlled. Irrigated tip catheter ablation was conducted using power-controlled mode, initially using dragging ablation with 30W on anterior and 20W on posterior wall applications. After 2018, SURPOINT technology was available, then 40W ablation lesions were performed point-by-point aiming the index of 550 on anterior wall and 400 on posterior wall.

When there was any esophageal temperature increase, radiofrequency (RF) application was interrupted, the power was reduced to 20 or 15W (according to the case) and RF was continued only after temperature normalization. When using point-by-point SURPOINT guided lesions, time of application was reduced aiming for an index of 300. When a linear probe was used, the sensor position was guided by fluoroscopy aiming for a closer position of the sensor to the area of ablation. A TEE probe was used to displace the esophagus, if its position was observed to be close to the vein. In some cases, esophageal temperature was monitored using an esophageal displacement strategy. The multielectrode system used was Circa, with the alarm set in two levels, 37.5ºC (warn) and 38ºC (risky), respectively, at the start of the procedure. If there was repeated esophageal temperature increase, RF were delivered with short duration (5- 10s) until achieving pulmonary vein isolation.

### Esophagogastroduodenoscopy (EGD)

The EGD was performed within seven days after ablation, most on the following day after the ablation procedure. Lesions observed in the post ablation EGD were categorized according to the Kansas City Classification (KCC) system,^10^ with additional description of bruises and traumatic lesions and emphasis in KCC 2b type lesion (ulcers), due to its higher risk of progression to perforation and fistula. If the patient presented a lesion on the first EGD, the patient was discharged and maintained on protons pump inhibitors (PPI) and sucralfate. A second EGD was recommended after another 7 days in patients presenting more severe lesions (KCC 2b). Our ablation and EGD follow-up strategy has been published elsewhere.^4^

### Statistical analysis

Continuous data were presented as mean and standard deviation or median and interquartile range in case of asymmetrical distribution. Categorical data were presented as absolute frequency and percentages. The occurrence of ulcer was described according to each qualitative characteristic first in a bivariate analysis to examine the relationship between two variables. The association was verified using chi-square test or likelihood ratio test, while quantitative characteristics were described according to the occurrence of ulcer and compared using unpaired t-Student test or Mann-Whitney test according to the probability distribution of the data. The joint model was tested using multiple logistic regression with the characteristics that in the bivariate analysis presented a descriptive level below 0.20 (p < 0.20). The occurrence of ulcer was described at each endoscopy in patients who repeated the examination and verified the change in the occurrence of ulcer between examinations using the McNemar test.

The software IBM-SPSS for Windows version 22.0 was used to carry out the analyses and the software Microsoft Excel 2013 was used to tabulate the data. Data were tested using the Shapiro-Wilk test of normality. The tests were performed with a significance level of 5%.

## Results

### Patients and procedure characteristics

Patient characteristics are presented in Table 1. A total of 823 ablation procedures with controlled EGD were performed at our institution between January 2016 and December 2022. Most patients were men, with a median age of 60 years old, and with paroxysmal AF. The mean left atrial diameter and mean left ventricular ejection fraction were 42.3±6.3mm and 61±8%, respectively.

**Table 1:** – Patients and ablation procedure characteristics

	Total
**Patients**	823
**Male**	588 (71.4)
**Age (years)**	60 (52. 68)
**Classification of AF**	
Paroxysmal	575 (69.9)
Persistent	248 (30.1)
**CHA2DS2-VASc score**	1.66±1.39
**LA diameter (mm)**	42.3 ± 6.3
**LA volume (mL/m^2^)**	39.5 ± 12.8
**LVEF, %**	61.3 ± 8.5
**Prior electric cardioversion**	189 (22.9)
**Prior AF Ablation**	147 (17.8)
**Electroanatomical Mapping**	655 (79.6)
**Ablation Aproach**	
Fluoroscopy	168 (20.4)
Carto 3	601 (73)
Ensite	53 (6.4)
Microport	1 (0.1)
**Ablation Strategy**	
50/30W, 55T	150 (18.2)
Dragging 30W	332 (40.3)
HPSD	40 (4.9)
SurPoint	261 (31.7)
Cryoablation	6 (0.7)
PVAC	34 (4.1)
**Catheter**	
8mm	149 (18.1)
ThermocoolST	135 (16.4)
STSF	469 (57)
Tacticath	27 (3.3)
Flexability	2 (0.2)
ArcticFront	6 (0.7)
PVAC Gold	34 (4.1)
3DFireMagic	1 (0.1)
**Esophageal Protection**	
No Device	24 (3.1)
Linear Probe	310 (40.3)
Multielectrode Probe	306 (39.8)
TEE Probe	122 (15.9)
Device Displacement	7 (0.9)
**Procedure duration (minutes)**	210 (180; 260)

Values are presented as N (%), mean±standard deviation or median (p25–p75). AF: atrial fibrillation; LA: left atrium; STSF: Thermocool Smarttouch Surroundflow; LVEF: Left Ventricular Ejection Fraction; TEE: Transesophageal Echocardiogram; PVAC: pulmonary vein ablation catheter.

Circumferential pulmonary vein isolation was the standard approach either with EAM or guided by fluoroscopy in patients from the public system. Most (91.8%) of the EAM cases were performed with Carto system and contact sensor catheters, mainly with the Smartouch Surround Flow (STSF) technology. The strategy used for ablation changed over the years and is described in Figure 1.

**Figure 1:** – Yearly strategy for atrial fibrillation ablation adopted at InCor; there was a change in ablation strategy as new technologies have become available (p<0.001).

Esophageal protection strategy could be applied in 769 cases. Single-sensor probe was the strategy used in 40,3% of the cases and multi-sensor in 39,8%. Transesophageal probe deviation was used in 15.9% of the cases, and specific device displacement in 0.9%. No device was used in only 3.1% of the cases, in which RF power on the posterior wall was empirically reduced. There was a change in the main esophageal protection strategy adopted over the years, which is described in Figure 2.

**Figure 2:** – Yearly distribution of strategies for esophageal protection in atrial fibrillation ablation adopted at InCor from 2016 to 2022 (p<0,001). In 2016 and 2017 the most common strategy was the linear probe. From 2017 to 2018 we started esophageal displacement using transesophageal echocardiogram probe, that was abandoned in 2019; after that, multielectrode S shaped probe was the most common strategy.

### Incidence of esophageal lesions

Acute abnormalities were detected in 26,2% of the 823 EGDs. Hematoma and ecchymosis were found in 3.3% of patients, erythema (KCC1) in 1.7% and erosion (KCC2A) in 9.5%. Esophageal lesions classified as KCC2B (ulcer) represented 8.2% of the cases. Traumatic esophageal lesions were found in 3.8% of patients in which the TEE probe was used to displace esophagus. The incidence of esophageal lesions following EGD is described in Table 2. In 277 (30.6%) endoscopies performed after ablation there was a significant amount of food remnants, despite fasting state of eight hours. This was attributed to gastric emptying reduction secondary to RF applications close to the esophagus and vagus nerve, leading to gastroparesis.

**Table 2:** – Endoscopic lesions detected in patients undergoing esophagogastroduodenoscopy

No Lesion	606 (73.5)
**Hematoma-Ecchymosis**	27 (3.3)
**Erithema (KCC1)**	14 (1.7)
**Erosion (KCC2A)**	78 (9.5)
**Ulcer (KCC2B)**	67 (8.2)
**Traumatic Lesion**	31 (3.8)

KCC: Kansas City Classification.

### Predictors of esophageal lesions

In a bivariate analysis, there was no difference in age, LA size or ejection fraction between patients with and without ulcer. Non-esophageal protective strategies were correlated with less KCC2B esophageal lesions. The median highest esophageal temperature was similar in both groups: 38.6 (38; 39.5) ºC in patients without ulcer and 38.7 (37.8;40)ºC in patients with ulcer. Among the various parameters of the bivariate analysis specified in Table 3, gender, LA size, type of previous oral anticoagulant, type of catheter, EAM, ablation strategy, and esophageal protection showed a descriptive level below 0.20 (p < 0.20) and were subsequently included in a multiple logistic regression analysis. In the logistic regression model, female gender was correlated with higher risk of ulcer. The use of an 8mm tip catheter also increased the risk of ulcer (Table 4).

**Table 3:** – Description of ulcer occurrence according to the studied population and results of bivariate analyses

Variable	No Ulcer	Ulcer	p value
**Age (years)**	60 (52; 68)	63 (50; 71)	0.578
**Gender**			
Male	546 (93.5)	38 (6.5)	0.006
Female	206 (87.7)	29 (12.3)	
**CHA2DS2-VASc score**			0.338
**Classification of AF**			0.643
Paroxysmal	526 (92)	46 (8)	0.825
Persistent	226 (91.5)	21 (8.5)	
**LA (mm)**	42.5 ± 6.3	40.9 ± 5.5	0.065
**LA (mL/m^2^)**	39.8 ± 12.9	37.1 ± 11.3	0.254
**LVEF (%)**	63 (60; 66)	65 (60; 68)	0.360
**Type previous DOAC, n (%)**			0.084
**Prior electric cardioversion**			0.504
**Prior AF Ablation**			0.786
**Maximum temperature (ºC)**	38.6 (38; 39.5)	38.7 (37.8; 40)	0.705
**Procedure duration (minutes)**	210 (180; 260)	210 (180; 270)	0.766
**Catheter, n (%)**			0.037
8mm	127 (85.2)	22 (14.7)	
ThermocoolST	124 (91.9)	11 (8.1)	
STSF	438 (93.3)	31 (6.7)	
Tacticath	27 (100)	0 (0)	
Flexability	2 (100)	0 (0)	
ArcticFront	6 (100)	0 (0)	
PVAC Gold	31 (91.2)	3 (8.8)	
3DFireMagic	1 (100)	0 (0)	
**Electroanatomical mapping, n (%)**	604 (92.8)	47 (7.2)	0.048
**Ablation Approach, n (%)**			0.243
Fluoroscopy	148 (88.1)	20 (11.9)	
Carto 3	556 (93)	42 (7)	
Ensite	47 (90.4)	5 (9.6)	
Microport	1 (100)	0 (0)	
**Ablation Strategy, n (%)**			0.038
50/30W, 55T	127 (85.2)	22 (14.8)	
Dragging 30W	305 (92.1)	26 (7.9)	
HPSD	38 (95)	2 (5)	
SurPoint	245 (94.6)	14 (5.4)	
Cryoablation	6 (100)	0 (0)	
PVAC	31 (91.2)	3 (8.8)	
**Esophageal Protection, n (%)**			0.122
No Device	21 (87.5)	3 (12.5)	
Linear Probe	279 (90.3)	30 (9.7)	
Multielectrode Probe	287 (94.4)	17 (5.6)	
TEE Probe	107 (88.4)	14 (11.6)	
Device Displacement	7 (100)	0 (0)	

Values are presented as N (%), mean±standard deviation or median (p25–p75). AF: atrial fibrillation; LA: left atrium; LVEF: Left Ventricular Ejection Fraction; STSF: Thermocool Smarttouch Surroundflow; TEE: Transesophageal Echocardiogram ; DOAC: oral anticoagulant.

**Table 4:** – Multivariate analysis of main factors related to the incidence of esophageal ulcer

Variable	OR	95% CI		p
		Lower	Upper	
**Female sex**	1.82	1.02	3.32	0.041
**Left atrium (mm)**	0.97	0.92	1.01	0.129
**Ejection fraction (%)**	1.02	0.98	1.06	0.318
**8mm Catheter**	3.14	1.21	8.18	0.019

Multiple logistic regression.

### Follow-up of esophageal lesions

In most patients with minor traumatic lesions, EGD was repeated during outpatient care and the result evaluated by the referring doctor. In the 67 patients with ulcer, the mean size of the lesion was 9.6±3.8mm. This lesion healed in 57 (83.8%), but 11 (16,2%) maintained KCC2B lesion. Six of them (9% of the cases with ulcer) were readmitted to the hospital, and the first computed tomography (CT) showed no evidence of fistula in these cases. Four of the patients were maintained on total parenteral nutrition, one on enteral nutrition through an endoscopy- positioned esophageal tube and other with cold soft diet, omeprazole and sucralfate. EGD was repeated in those not presenting signs of fistulization in a new CT scan. One patient progressed to esophageal-mediastinal fistula 14 days after ablation. The procedure was performed with an 8mm tip catheter, fluoroscopy guided. The patient was successfully treated endoscopically with metal clip and endoloop technique (Figure 3).

**Figure 3:** – Progression of patients with ulcer. EGD: esophagogastroduodenoscopy; CT: computed tomography.,

## Discussion

Esophageal lesions are still a problem during AF ablation procedures. Despite rare, with incidence lower than 0.02%, these lesions require protective measures. In the past years, new esophageal protective technologies and ablation techniques have led to different procedural risks.^11,12^

A prior Brazilian national survey that included 10 AEF cases from eight centers with 8603 ablation procedures between 2003 and 2015, resulting in an incidence of 0.113%.^3^ In this series, half of the patients presented clinical manifestations of AEF that were not noticed by their physicians. Additionally, seven of the 10 patients died and only one had a complete recovery. Because of those findings we decided that all patients in our group who underwent AF ablation should undergo endoscopic surveillance allowing to early detect patients at risk and to initiate early treatment when appropriate.^4^

We routinely perform endoscopy on the day following AF ablation procedure. All patients regardless of endoscopy findings are maintained with PPIs for 30 days. For the patients with low-risk lesions (erythema and small superficial erosions), we maintain only PPI treatment without a control endoscopy. Patients with erosions larger than 10mm, ulcers or bullous lesions, endoscopy is repeated seven days later. Additionally, sucralfate is added to increase esophageal and stomach acidity. When ulcers still persist seven days later, patients are hospitalized, maintained under fasting state and intravenous PPIs, and both antibiotics and anticholinergic drugs are initiated. Esophageal CT with oral contrast is performed to search for esophageal perforation and repeated a few days later before repeating a CO2 inflated endoscopy. In patients in whom ulcer still is increasing and those with any sign or symptoms of esophageal perforation endoscopic interventional treatment (Figure 4) is performed.^13,14^

**Figure 4:** – Protocol of evaluation and treatment of esophageal lesion. EGD: esophagogastroduodenoscopy; CT: computed tomography.

Recently we published our series of 677 AF patients that underwent EAM-guided RF ablation, PVAC or cryoablation.^9^ In the present series we included those patients, but we also included 149 patients that underwent RF using 8mm tip catheters. In the prior series, severe lesions (KCC 2B) were found in 6.8% of the patients.

In the present series, esophageal ulcer was detected in 67 of 823 cases (8.2%). Esophageal lesions healed in most of the cases after one week. Six patients were readmitted to the hospital and were maintained in parenteral nutrition, post-pyloric enteral nutrition or soft diet following the protocol. A chest CT and new EGD were performed and one patient developed esophageal fistula (Central Illustration). This patient underwent pulmonary vein isolation using the 8mm tip catheter. After admission to the hospital, a CT seven days after ablation revealed parietal thickening and slight densification of the mediastinal adipose tissue adjacent to the middle third of the thoracic esophagus, without signs of pneumomediastinum or fistula. The patient was kept fasting and with parenteral nutrition. A new CT 14 days after the ablation showed a small lesion in the left anterior wall of the middle third of the thoracic esophagus, associated with a tiny air bubble of pneumomediastinum near the posterior wall of the left atrium. It was decided to perform endoscopic treatment with clips and endoloop technique.^14^ This strategy is indicated for hemostasis in gastrointestinal bleeding and esophageal tumors; its use in case of fistula following AF ablation is not well-documented in literature. Nonetheless, as we could observe the first signs of fistula formation and since the patient had no fever or evidence of systemic infection in blood tests, it was decided to perform the endoscopic treatment available and prophylactic antibiotic for 14 days.

New controlled CT was performed on the fourth day after the endoscopic treatment. Enteral nutrition was initiated on the sixth day. A new CT was performed three weeks after the endoscopic treatment with no signs of fistula. After ruling out remaining fistula, a new EGD revealed a scar retraction in the esophagus and closure of the fistula. Then the patient initiated an oral diet and was discharged home. There were no more complications in a one-year follow-up. The other five patients that were hospitalized had their ulcer healed in sequential EGD and would probably have presented a worse outcome if the risk of perforation had not been identified and clinical treatment prescribed.

Prior studies reported an incidence of any esophageal lesions in control endoscopy ranging from 1.6%^15^to 35.5%.^8^ There are several differences among these studies, regarding the esophageal temperature monitoring strategies, the use of protection devices and the biophysics of the RF applications. Yarlagadda et al.^10^ presented an incidence of 570 lesions (15%), 206 (36%) type 1 lesions (KCC 1), 222 (39%) type 2a (KCC 2A) and 142 (25%) type 2b (KCC 2B). Six of 142 KCC2B (4.2%) progressed to type 3: five were type 3a and one was type 3b. In their series, thow patients died, one that presented type 3a and other type 3b. In this series only patients with initial KCC2B lesions progressed to fistula. Halbfass et al.^8^ identified a prevalence of 18% of any lesion and 6% for ulcerated lesions in asymptomatic patients. Different from Halbfass et al.,^8^ in our series, EGD was systematically performed, possibly improving detection accuracy for subclinical lesions, resulting in higher incidence rates.

A recent multicenter survey, the POTTER-AF study, reported a total fistula incidence of 0.025%.^16^ However, the actual incidence might be higher, as most centers have not implemented routine post-procedural detection protocols, leading to potential underdiagnosis and underreporting.^17,18^ The absence of AEF cases among patients from 1996 to 2003 could be attributed to differences in ablation techniques, poor detection, and insufficient active screening. At our institution, we have implemented targeted measures to identify at-risk patients. In the survey, the highest number of AEF cases reported by a single center was five, with a maximum incidence of 0.4% within a specified timeframe. This highlights the variability in diagnostic accuracy across participating centers. Consistent with findings from a previous Brazilian survey,^3^ the POTTER-AF study showed that only patients with early diagnosis and prompt referral for surgery survived.

Reduced gastric emptying, evidenced by the presence of food remnants despite fasting, was observed in 32% of cases. In the literature, symptomatic gastric motility alterations resulting from thermal injury to the periesophageal neural plexus have been reported in 5% to 74% of procedures. In some cases, a transient but severe gastric palsy may occur, requiring prolonged fasting.^19^

Interestingly, in our series, the female gender was independently related to higher risk of esophageal lesions. This could be related to the finding that women usually present a thinner LA wall protecting the esophagus.

An independent factor associated with a higher incidence of esophageal lesions in this series was the use of 8mm tip catheters (OR 3.14, CI 1.21–8.18; P=0.019). This likely contributed to the increased incidence of esophageal lesions compared to our previous publication.^9^ Although 8mm tip catheters have been largely abandoned in favor of irrigated tip catheters in most centers, we continue to use them as they can be reprocessed, enabling us to offer AF ablation to patients in the public healthcare system. So when those catheters are used, additional surveillance must be implemented.

In our series, the approach to esophageal protection during ablation has changed over the years. Single-sensor and multi-sensor esophageal temperature monitoring probes were used in most cases. Until 2018, esophageal deviation with a TEE probe was employed in more than half of the procedures; however, this strategy was discontinued due to significant mapping distortions and the risk of traumatic lesions.^20^ Currently, the S-shaped multi-sensor probe is our primary protective strategy. Previous studies on esophageal cooling as a protective measure against fistula formation have yielded mixed results.^21^ More recently, the IMPACT study^22^ demonstrated that thermal protection strategies aimed at reducing esophageal temperature are safe and can significantly decrease ablation-related thermal injuries.

Different to prior reports,^23^ the use of high-power short-duration pulses has not been correlated with less esophageal lesions. Similarly, the use of contact force and SurPoint technology have not proved to be safer than other approaches. New technologies such as pulsed-field ablation are promising, but not yet universally available. Thus, the concern with esophageal injury is still a reality.^24-27^

The main limitation of this study is that this is a retrospective data recording of a strategy that was implemented in 2016 in our patients who underwent a control EGD after AF catheter ablation, despite the finding of esophageal temperature increase. Most of the first EGDs could be recorded since it was performed on the day following ablation. Only patients presenting more severe lesions underwent EGD one week later. Recent observation has suggested that early endoscopy may fail to detect severe lesions that appear in the following weeks.^26^ However, none of our patients returned to the hospital for esophageal lesion treatment, nor presented any complication in the follow-up.

Although the use of 8mm tip catheters is considered an “old” strategy, it is still employed in some centers. In our series, their use was associated with a higher risk of esophageal lesions. From our perspective, this is not a limitation of the study but rather an important finding to raise awareness among physicians about the increased risk associated with these catheters.

Our findings should be interpreted as hypothesis-generating and warrant further investigation in prospective studies.

## Conclusions

The incidence of esophageal lesions after AF catheter ablation when routine endoscopy is performed is high, but in most of the cases, the lesion is spontaneously healed. However, ablation approach with 50/30W using 8mm tip catheter is related to more ulcers, and a better esophageal protective strategy is still to be defined. Early esophagus endoscopy may identify patients at risk for AEF after AF ablation, and early clinical treatment seems effective to prevent progression and worse clinical outcomes.
